# Lower Body Joint Angle Prediction Using Machine Learning and Applied Biomechanical Inverse Dynamics

**DOI:** 10.3390/s23010228

**Published:** 2022-12-26

**Authors:** Zachary Choffin, Nathan Jeong, Michael Callihan, Edward Sazonov, Seongcheol Jeong

**Affiliations:** 1 Department of Electrical and Computer Engineering, The University of Alabama, Tuscaloosa, AL 35487, USA; 2 Capstone College of Nursing, University of Alabama, Tuscaloosa, AL 35487, USA; 3 Department of Electrical Engineering, Pohang University of Science and Technology, Pohang 37673, Republic of Korea

**Keywords:** foot sensor, joint angle detection, lower body, machine learning, inertial measurement unit

## Abstract

Extreme angles in lower body joints may adversely increase the risk of injury to joints. These injuries are common in the workplace and cause persistent pain and significant financial losses to people and companies. The purpose of this study was to predict lower body joint angles from the ankle to the lumbosacral joint (L5S1) by measuring plantar pressures in shoes. Joint angle prediction was aided by a designed footwear sensor consisting of six force-sensing resistors (FSR) and a microcontroller fitted with Bluetooth LE sensors. An Xsens motion capture system was utilized as a ground truth validation measuring 3D joint angles. Thirty-seven human subjects were tested squatting in an IRB-approved study. The Gaussian Process Regression (GPR) linear regression algorithm was used to create a progressive model that predicted the angles of ankle, knee, hip, and L5S1. The footwear sensor showed a promising root mean square error (RMSE) for each joint. The L5S1 angle was predicted to be RMSE of 0.21° for the X-axis and 0.22° for the Y-axis, respectively. This result confirmed that the proposed plantar sensor system had the capability to predict and monitor lower body joint angles for potential injury prevention and training of occupational workers.

## 1. Introduction

Millions of US workers go to work every day with an expectation of returning home safely at the completion of their shifts. Despite immense efforts in mitigation against musculoskeletal injury, 26.1 out of every 10,000 workers in the United States experience a musculoskeletal injury every year [1]. The numbers have been improved through the National Institute of Occupational Safety and Health (NIOSH) hierarchy of controls [2] for injury mitigation. As of 2019, injuries remain at 2.6 out of every 100 workers, The Bureau of Labor Statistics (BLS) reports this number is only down 13% since 2014 [3]. Numbers have gone down since 2019, which can be attributed to the COVID-19 pandemic and its subsequent reduction of the workforce. To fully achieve the potential of the current mitigation efforts, the worker must be able to achieve proper body positioning while performing their work [4].

Aside from injuries in the workplace, people are constantly bending, twisting, walking, and lifting things throughout the day. During these movements, a ground reaction force is created between the ground and the bottom of the feet. This force is then transferred up the body through the ankles and knees, and then ultimately into the hips and lower back [5]. When the lower extremities perform these tasks in an awkward positioning, the force is increased in the joints, which in turn increases the risk of injury. Real-time monitoring to identify these awkward positions allows for directed interventions to improve the body mechanics people utilize, thus reducing their risk of musculoskeletal injury [6].

Multiple technologies have been employed to measure joint angles of the human body. Table 1 summarizes various joint angle detection methods. IMU-based motion capture systems, body markers, surface electromyography (sEMG), and ultrasonic imaging have been used primarily to predict full body joint angles and motion. Joint angles are often measured in a laboratory setting [7,8,9,10,11,12], providing a highly accurate system with limited mobility. IMU-based systems [13,14,15] and sEMG [16,17,18,19,20,21,22,23] systems allow for high accuracy with reasonable portability. However, these systems may require complex calibration procedures and are difficult to use in daily life [24,25].

To increase mobility and maintain accuracy for daily use, simpler and practical sensor systems are required [26,27]. Research into completely mobile systems have demonstrated the feasibility of achieving both high accuracy and mobility in the same system. Shoe-based sensor systems present a discrete and accurate solution capable of tracking gait patterns to measure joint angles [15,26,27,28,29,30,31,32,33]. In our previous study, a force-sensing resistor (FSR)-based plantar pressure sensor system (P2S2) was developed to predict the ankle angles [27].

When a human body interacts with the ground, it creates a force known as the ground reaction force. This is a variable force that depends upon the weight and position of the person. This force is distributed from the foot into the ankle and follows up the rest of the body. When in contact, the position of the foot relative to the ground influences the distribution of pressure throughout the body. The sensor system designed for this study uses six FSR sensors that were placed at common pressure points.

Prediction of the joint angles along the kinematic chain of the lower body can be accomplished by applying the principles of inverse dynamics presented by Winter [5]. In this paper, we present a proof of concept of predicting the two-dimensional angles of the ankle, knee, hip, and at L5S1 in the sagittal plane using a machine learning algorithm and biomechanical inverse dynamics. Abduction and adduction are tracked consistently between the Xsens motion capture system and the FSR to expand on this proof of concept, while rotation about the joints is not addressed.

This system proposes an accurate, wearable mobile platform, which is a relatively comfortable, non-invasive, cost-effective, and affordable alternative to real-time monitoring of lower body joint angles.

## 2. Materials and Methods

### 2.1. Footwear Sensor

The common pressure points were determined according to previous studies [34,35,36], and our separate internal test was conducted with 40 participants wearing shoe sizes ranging from size 3 to 15. Two sensors are placed below the talus of the foot, three sensors are placed along the heads of the metatarsals, and the last sensor is placed on the first distal phalanges. The sensors are placed under the insole and are difficult to feel for the wearer. While this is still a prototype and further development is needed, the insoles are relatively comfortable to wear. When an FSR experiences a mechanical stress perpendicular to the sensor plane, the resistance changes, following a predictable logarithmic curve. This variance was used to estimate the force applied to an individual sensor. The resistance of the sensor varies between 30 k Ω and 10 MΩ, depending on the force applied. The FSR sensors were designed and manufactured by Tekscan [37].

**Figure 1:**
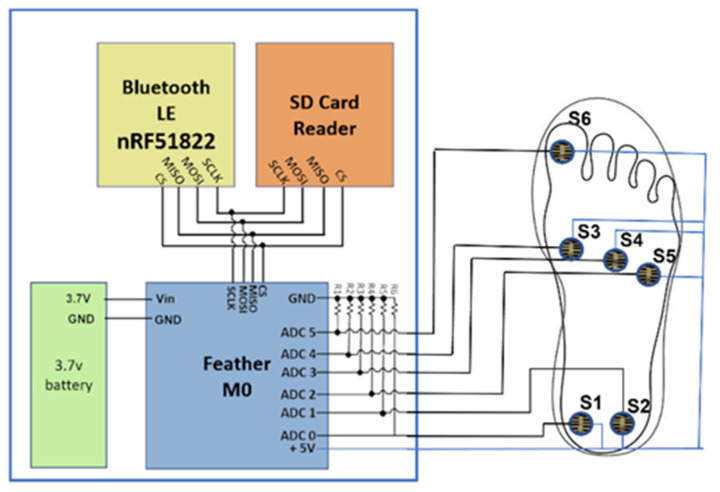
Circuit schematic of the proposed footwear sensor system attached to the right foot.

This sensor system is combined with a commercially available microcontroller equipped with an added Bluetooth Low-Energy Module (Adafruit Feather M0 Bluefruit LE [38]) and an SPI-controlled SD card reader [39]. Figure 1 shows a circuit schematic of the proposed footwear sensor system. The microcontroller used on the Feather M0 is an ATSAMD21G18 ARM Cortex processor. This features a clock frequency of 48 MHz and 3.3 V logic. The ARM processor features 356 KB of flash memory and 32 KB of RAM. Using the six channel 10-bit analog-to-digital converter (ADC) connected to each FSR, the analog voltages can be converted to digital values to represent each sensor’s resistance. The Bluetooth LE was realized using the nRF51822 chipset from Nordic. A communication script was written in the Arduino IDE to communicate with the controller via a Universal Asynchronous Receiver Transmitter (UART) connection scheme. This implementation saves pressure-sensing data to the SD card for post-data processing. A smartphone is used in combination with the Bluetooth LE chip to control the starting and stopping of tests remotely. Then, 10 KΩ resistors are used in a pull-down configuration to ground as a reference. The insole itself uses a flexible substrate composed of cellulose acetate. The wires used are 23 American Wire Gauge (AWG) solid copper wires, which provide a flexible yet stable connection. An additional layer of cellulose acetate was used to protect the wires and FSRs from shear forces that can develop from the shoe’s insole. The data of the insole sensors are recorded at 50 Hz, then stored on the SD card in real time. The data are then used in post-processing and feature extraction to predict lower body angles.

**Figure 2:**
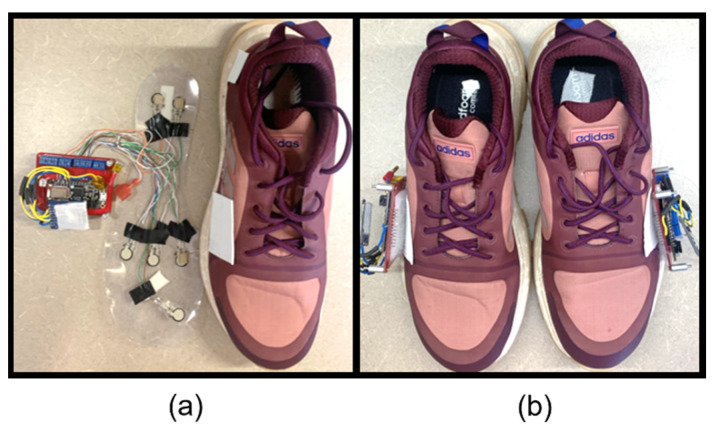
Complete footwear sensor system for women: (**a**) women’s size 8.5 with P2S2s and (**b**) a pair of shoes assembled for testing.

**Figure 3:**
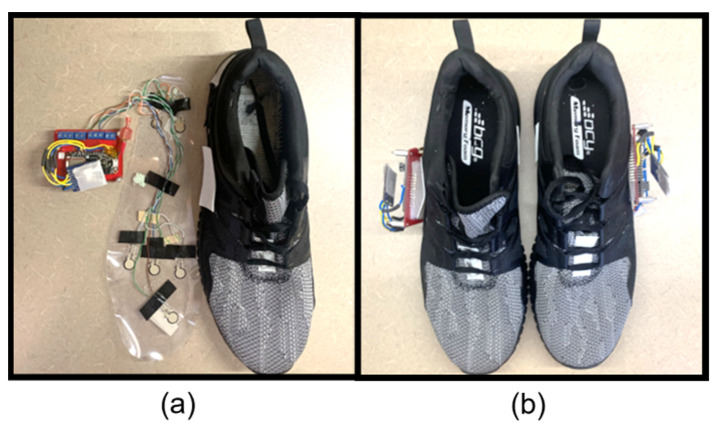
Complete footwear sensor system for men: (**a**) men’s size 10.5 with P2S2s and (**b**) a pair of shoes assembled for testing.

To examine the greatest number of participants possible for our study, two shoes of different sizes were developed. The shoe sizes used were based upon a cross-reference study [40], which reported the most common US shoe sizes as 10.5 for males and 8.5 for females. The complete insole systems inside the shoes are displayed in Figure 2 and Figure 3. The wires were fed through a slot cut into the shoe and directly attached to the PCB adhered to the side of the shoe.

### 2.2. Reference Data Collection System

Reference data of human joint angles are collected using the Xsens motion capture system [41]. Seventeen IMUs are attached to the participant at the manufacturer’s recommended locations and secured with the appropriate Velcro straps. Calibration of the system is completed with the participant standing with their feet together and looking forward with their hands down to their side (i.e., n pose). The participant then walks forward and returns to the starting position. Following walking calibration, the model is applied to the participant and all movement is verified with the visualization feature of the Xsens software program. The orientations of the IMU devices were recorded in the proprietary software and transferred as an MVNX file for processing in the Visual 3D ver. 2021x64 software package as three-dimensional (3D) angles [42].

While it is known that accuracy of the IMU system is questionable for abduction/ adduction (y-axis), the movements of flexion and extension (x-axis) have been found to be relatively accurate [42,43]. For this proof-of-concept study, the Xsens system was deemed adequate [44,45,46] by the research team to demonstrate the sensors’ ability to track movements consistently. To further improve accuracy of this project, validation using skin markers and an optical camera system will be conducted in the future.

### 2.3. Experimental Procedure

**Figure 4:**
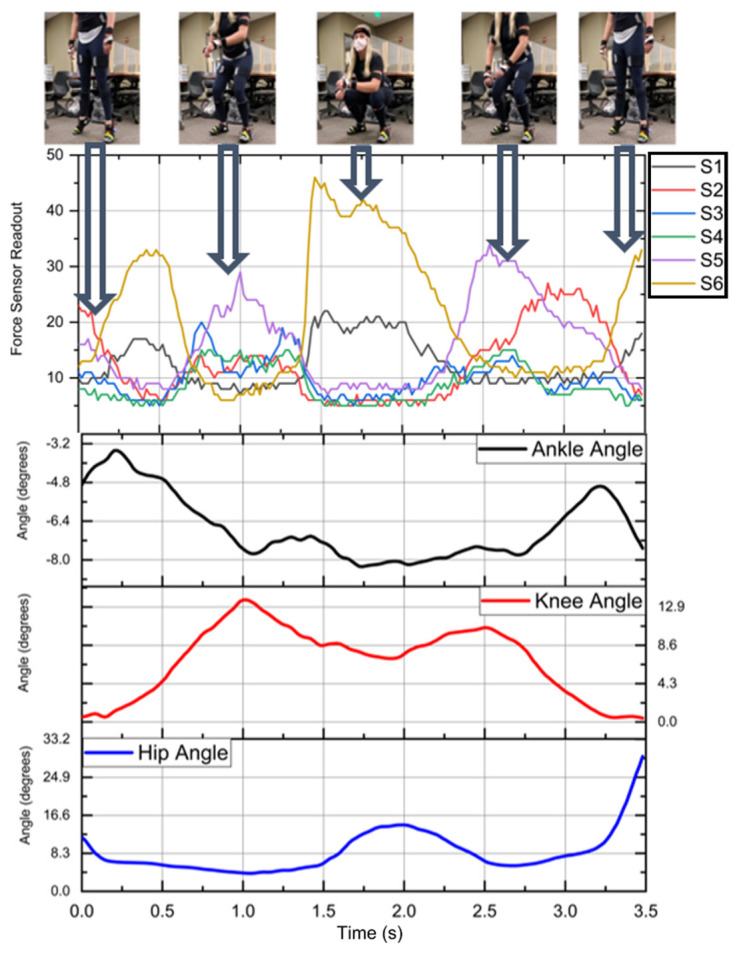
Example of squat motion with accompanying FSR data and Xsens angle outputs.

Squatting is a movement commonly used to lift objects in a work environment [47]. The squatting motion in this study was performed using a standardized squatting position [48]. The squatting position of this study required the subject to enter a shallow squat that descended no lower than parallel to the ground. After reaching the bottom of the squat, the subject would rise and hold a standing position for a moment. Then, they would complete the motion with a heel raise. This motion was repeated a total of 10 times per participant. Example data of the squat movement and its accompanying sensor readout are shown in Figure 4. As the participant raises their heels and the back of the shoe lifts off the ground, S1 and S2 fall to zero. S3 to S6 rise as the weight shifts to the front of the shoe, then followed the heels hitting the ground. S1 and S2 both report a significant rise in pressure that then is reduced as the subject enters into a squat. When the subject returns to a standing position, S1 and S2 fall, and then proceed to rise. This signals the end of the squat motion. At the beginning and end of each motion, a heel raise is performed. Body movement manipulation has proven to be effective at synchronization of independent systems [49,50]. To account for Bluetooth interference, motion data containing a sampling rate beyond a 50 Hz ±2 Hz margin are removed from the dataset.

This study is authorized by the Institutional Review Board (IRB NO. 20−02−3356−A) at The University of Alabama. In total, 37 participants including 26 females and 11 males were recruited for the study from the college of nursing and engineering. In total, 26 of the participants wore a size 8.5 shoe, and they were 1.66 ±0.049 m in height, 64.3 ±8.12 kg, and 21.2 ±1.79 years in age. The other 13 participants wore a size 10.5 shoe with height 1.80 ±0.054 m, mass 73.5 ±8.39 kg, and age 21.4 ±1.15 years. The sanitized data of all participants who contributed to the study are displayed in Table 2.

The testing location selected was The University of Alabama’s Capstone College of Nursing. Upon arrival, informed consent was obtained from all participants, screening for COVID-19 exposure was conducted by a registered nurse, and secondary screening for inclusion was performed by a member of the research team. Participants were included if they wore shoe sizes of 10.5 or 8.5 and did not have existing musculoskeletal problems of the lower extremities. Following consent and screening procedures, participants were fitted with the Xsens motion capture system and the FSR system. Participants were then asked to complete fourteen movements, one of which was squatting. Collection time for the study ranged from 45 min to 1 h for each participant.

## 3. Machine Learning to Predict Lower Extremity Angles

**Figure 5:**
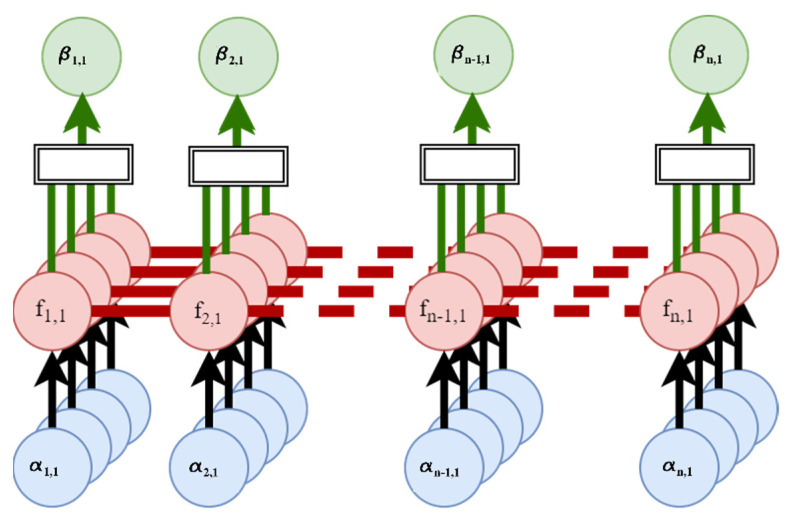
Exponential Gaussian Process Regression model used to predict ankle, knee, hip, and L5S1 angles.

Data gathered during the study contains a level of randomness and nonlinearity due to the inconsistency of human subject movements. To overcome the human factor, this study employs a supervised non-parametric Bayesian regression learner, GPR, for better prediction of human joint angles. Human data do not present isotropic behavior; when humans step the pressure may be the same, but movements differ. In the proof-of-concept nature of the research, a more computationally expensive algorithm is utilized. Given a GPR’s ability to use probability in calculations, this improves the model’s ability to predict joint angles. Probability allows for the algorithm to determine lower body joint angles when similar patterns are present. Figure 5 shows the general process diagram of the GPR’s function. The exponential GPR is chosen based on MATLAB’s regression learning classifier. MATLAB is beneficial for the creation of machine learning algorithms, as it allows for rapid design and testing. The GPR creates a collection of finite number time variables. In the GPR function, data are fed through at each sample containing *α* number of features. The features pass through the Gaussian field parameters and estimate the output based on the features in *f*. The results of each element are summed together in the form of the output *b*.

**Figure 6:**
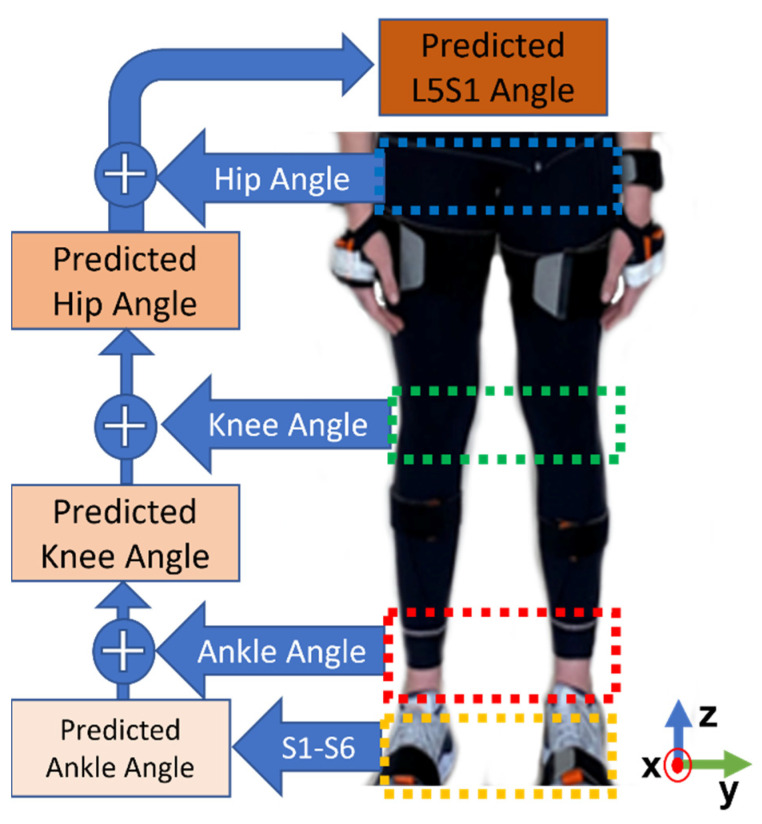
Applied inverse dynamics approach to predict lower body joint angles.

All participants’ squat data are gathered and post-processed to extract features. The feature set changed for each joint angle model; Figure 6 displays the flow chart of feature design. The features used in the ankle angle prediction model are the six FSR sensor outputs. Using the bottom-up approach of inverse dynamics, plantar pressure sensor data can be transformed into lower body joint angles. In other words, the knee joint prediction included the initial six FSR sensors’ data and the predicted ankle angle in each axis of movement. The hip angle follows the same procedure, but also includes the predicted ankle and knee angles. In L5S1 calculation, the feature set includes FSR sensors’ data and ankle, knee, and hip angles in each axis.

The data of 33 participants are used to train the GPR model. The data of four participants are completely isolated to validate the machine learning model. A 10-fold cross-validation is used to train the model and detect instances of overfitting. This results in a total of 57,480 points used in the training and validation of the model. Further, 4920 data points are used for testing of the model between four participants. Each test result’s RMSE is then calculated based on the model-predicted data.

(1) $$RMSE=\sqrt{\overset{n}{\underset{i=1}{\sum }}\frac{{\left(\hat{y_{i}}-y_{i}\right)}^{2}}{n}},$$

where $\hat{y_{i}}$ is predicted value of the model, $y_{i}$ is the ground truth value from Xsens, and *n* is the sample size of the data. The equation outputs the average distance between the predicted linear regression lines from a line of best fit.

## 4. Results

The L5S1 joint angle was first predicted by only using the first six insoles to determine if inverse dynamics were necessary for joint angle detection without the L5S1 joint angle. For this testing, Participant 25 was removed from the model’s training set. The participant was selected randomly to reduce researcher bias on data. When tested with purely six sensors, the models produced an RMSE of 5.61 and showed a poor correlation to the L5S1 joint. This demonstrates the need to alter the testing approach to include more robust features.

**Figure 7:**
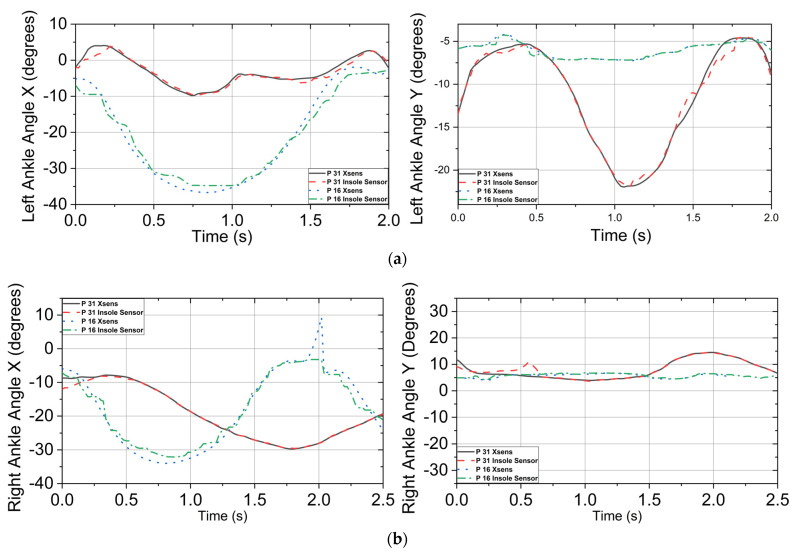

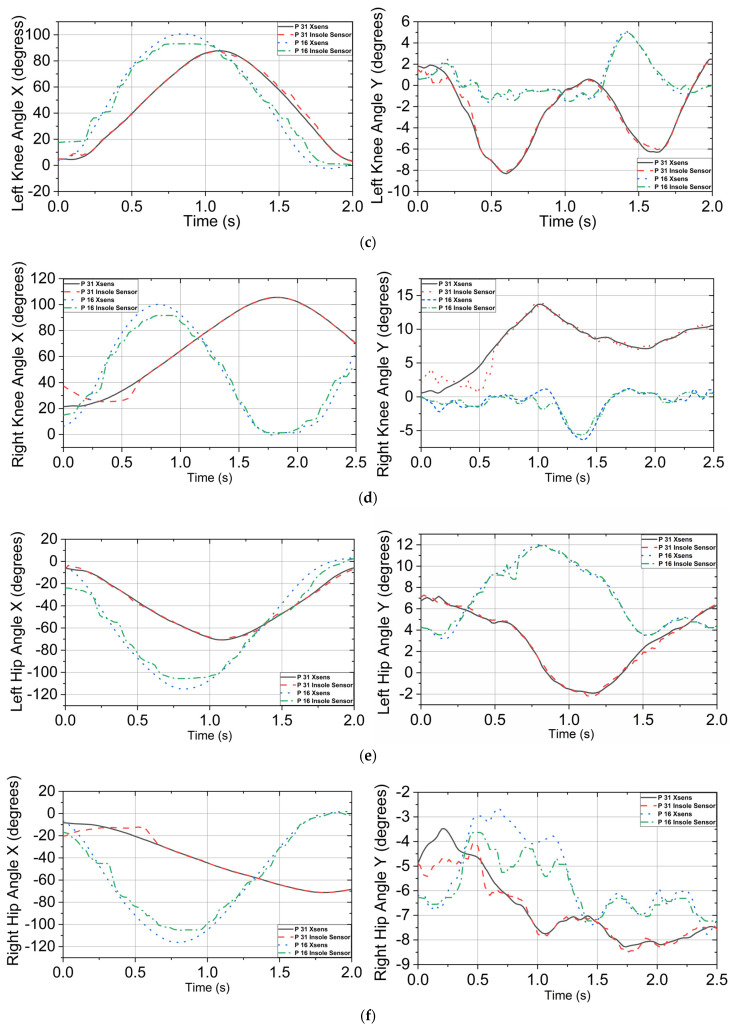

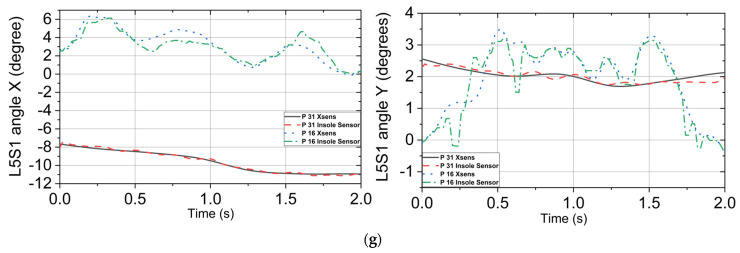
Joint angle detection in the X and Y axes from the ankle to the L5S1 joint, which is located above the pelvis for participants 31 and 16. (**a**) Left ankle angle actual vs. predicted in the X−axis and the Y−axis. (**b**) Right ankle angle actual vs. predicted in the X−axis and the Y−axis. (**c**) Left knee angle actual vs. predicted in the X−axis and the Y−axis. (**d**) Right knee angle actual vs. predicted in the X-axis and the Y-axis. (**e**) Left hip angle actual vs. predicted in the X−axis and the Y−axis. (**f**) Right hip angle actual vs. predicted in the X−axis and the Y−axis. (**g**) L5S1 angle actual vs. predicted in the X−axis and the Y−axis.

Figure 7 displays the result of a comparison between the predicted and measured joint angles. Participant 31’s 10th squat and participant 16’s 4th squat were randomly chosen to visualize the predicted joint angles. Figure 7a displays the left ankle in both the X and Y−axes over the duration of the movement. This movement versus the Xsens angle had an RMSE of 1.133° in the X−axis and 1.09° in the Y−axis for participant 31; participant 16 had an RSME of 2.2711° for the X−axis and 1.20° for the Y−axis. The right ankle angles are shown below in Figure 7b. This model encountered difficulty in processing the first section of the squat but tracked well after the first 3 s of the movement. The RMSE of these graphs was 0.639° for the X−axis and 1.13° for the Y−axis for participant 31; participant 16 had an RSME of 2.37° for the X−axis and 0.96° for the Y−axis. With the ankle angle models done, the predicted results of the model were placed into the squat data for the next knee angle prediction. Figure 7c displays the results of the model compared to the measured knee angle provided by the Xsens. This model achieved an RMSE of 0.81° on the X−axis and 3.77° on the Y−axis for participant 31; participant 16 had an RSME of 4.12° for the X−axis and 0.75° for the Y−axis. Figure 7d displays the predicted right knee angle over the duration of the squat versus the actual Xsens angle in X and Y-axes. The right knee had an RMSE of 3.29° on the X−axis and 0.99° on the Y−axis for participant 31; participant 16 had a RSME of 4.51° for the X−axis and 0.81° for the Y−axis.

These data were added to the squat for hip angle prediction, including the ankle prediction and FSR sensors’ outputs. Figure 7e shows the predicted hip angle plotted against the measured angle. The RMSE was 1.41° for the X−axis and 0.78° for the Y−axis for participant 31; participant 16 had an RSME of 3.61° for the X−axis and 0.73° for the Y−axis. Figure 7f displays an RMSE of 2.93° for the X−axis and 0.51° for the Y−axis for participant 31; participant 16 had an RSME of 3.54° for the X−axis and 0.79° for the Y−axis. Lastly, the L5S1 was predicted using a combination of all previous predicted angles and pressure sensor data. Figure 7g displays the L5S1 joint in the X and Y−axis. The RMSE of the X− and Y-axes was 0.21° and 0.22°, respectively, for participant 31; participant 16 had an RSME of 0.54° for the X-axis and 0.93° for the Y-axis.

**Figure 8:**
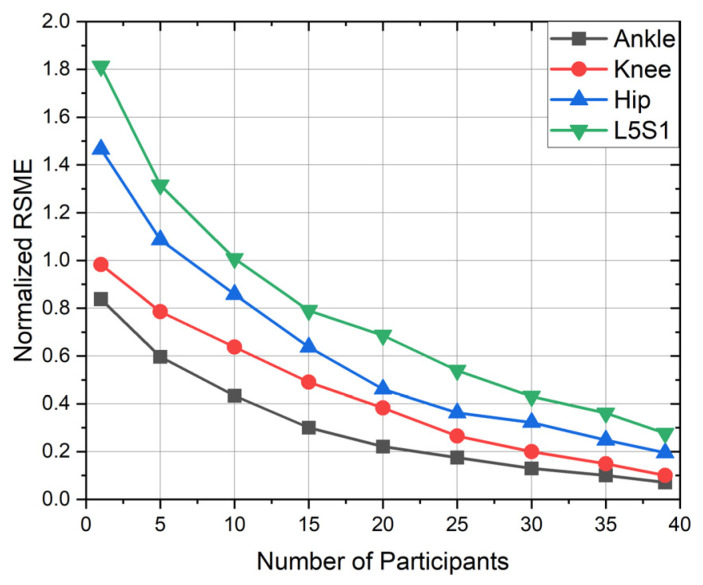
Model performance in terms of RSME as a function of the number of participants.

Figure 7 presents high correlation between the Xsens motion capture system and the P2S2 prototype with RMSE values ranging from 0.21° to 3.29° across the X−axis and 0.22° to 3.77° for the Y−axis. Data were filtered using the MATLAB Signal Analyzer Toolbox, specifically the smoothing filter with a smoothing factor of 0.2, which increased the overall accuracy of the joint detection. The effect of the number of subjects on accuracy is shown in Figure 8. These data were taken from the right foot in the X direction.

The possibility of the reduction sensor count is examined by activating and deactivating specific sensors during training. Test 1 includes sensors 2 and 5, which measure linear movement along the flexor digitorum brevis portion of the foot. Test 2 includes sensors 3, 4, 5, and 6 and represents two-dimensional movement excluding the plantar aponeurosis. Test 3 includes sensors 1, 3, 4, and 6, providing linear movement without the flexor digitorum brevis portion of the foot. Test 4 includes sensors 1 and 2 combined, which simulates a linear motion along the plantar aponeurosis. Test 5 utilizes all sensors except sensor 4 and represents motion from the second dorsal interosseous muscle. Test 6 utilizes all six sensors, showing two-dimensional movement in the mid foot and heel portion of the foot. The testing of each model was conducted on the same squatting motion of participant 25. Table 3 displays a summary of tests completed and their respective RMSE values.

## 5. Discussion

The proposed footwear-based pressure system with six FSRs was presented to predict the lower body angles from the ankle to the L5S1. This sensor system and the application of inverse dynamics could predict participants’ lower body joint angles with high accuracy relative to the Xsens motion capture system when performing the squat movement. While it is known that the accuracy of the IMU-based system for the abduction and adduction movements of the lower extremities is questionable during dynamic movements [42,43], the capability of the P2S2 to track movements consistent to the commercially available system was the purpose for this proof-of-concept study. The research team used the IMU system to identify the orientation of body segments to build a model within a second software package, Visual 3D, to ensure consistent data processing [42]. However, the rotation of the joints was out of the scope of the current study.

This study initially looked at participants between 19 and 39 years old, but more diverse ages would like to be studied. Although the P2S2 provides high correlation with the commercially available motion-capture system, the P2S2 does present some drawbacks. It cannot predict the angle of hip and L5S1 when one or both feet are off the ground, such in walking or running movements, due to the lack of a ground reaction force required for calculations. This limitation does not allow for the system to predict any angles during the swing phase of the gait cycle for the swing leg. IMU sensors placed with the P2S2 could improve joint angle tracking during a gait cycle.

Furthermore, our future study would like to look at additional movements to examine the capability of the system. This study used GPR to predict lower body joint angles; in the future, various machine-learning algorithms will be tested to explore potential improvement for a less computationally expensive but accurate model. Instead of Bluetooth for wireless data collection, Wi-Fi can be used to accommodate more participants in the test due to higher capacity of wireless connectivity and facilitate data transfer from a worker to healthcare provider.

In the current configuration of the P2S2 system, it has a total weight of 71 g, with 46 g on the outside of the shoe and 25 g inside the shoe. For the future, an all-in-one system is being developed to allow for the placement of all necessary electrical components below the insole. This design will decrease the weight of the system and increase its adaptability to the shoe industry. Additional shoe sizes will be developed to further generalize the machine learning model.

Using the proposed method, workers will have greater access to health-related biomechanical data, which may decrease the likelihood of workplace injuries. By measuring more complex movements, a customized shoe-based sensor system will be able to improve foot work of athletes in training.

## Figures and Tables

**Table 1:** Literature review of joint angle detection methods.

Author	Joint Angle	Sensor	Angle Measured	Mobility	Accuracy	RMSE
Jahanandish et al. [7]	Lower body	Ultrasonic imaging	3-D	Low	100%	N/A
Pang et al. [16]	Lower Body	sEMG	3D	Medium	90%	N/A
Coker et al. [17]	Knee	sEMG	3D	Medium	N/A	0.7
Shi et al. [18]	Lower body	sEMG	3D	Medium	N/A	2.0
Dey et al. [9]	Knee	Body markers	3D	High	99.5%	N/A
Dey et al. [8]	Knee	Body markers	3D	High	N/A	0.97
Sy et al. [14]	Lower body	IMU	3D	High	N/A	5.93
Zhu et al. [26]	Elbow	Resistive fibers	3D	Medium	N/A	N/A
Little et al. [23]	Elbow	sEMG	3D	High	N/A	N/A
Davarzani et al. [33]	Robotic joint	Capacitive plate	3D	Low	N/A	3.63
Choffin et al. [27]	Ankle	FSR	3D	High	93%	N/A

**Table 2:** Participant information.

Subject	Age	Sex	Height (m)	Weight (kg)	Shoe Size
1	21	Female	1.6	54	8.5
2	21	Female	1.63	84	8.5
3	21	Female	1.7	59	10.5
4	21	Female	1.7	61	8.5
5	21	Male	1.8	82	10.5
6	21	Female	1.75	77	10.5
7	21	Female	1.73	57	8.5
8	21	Female	1.63	75	8.5
9	21	Male	1.85	77	10.5
10	20	Female	1.7	63	8.5
11	24	Male	1.78	84	10.5
12	21	Male	1.8	77	10.5
13	20	Female	1.7	77	8.5
14	29	Female	1.6	66	8.5
15	23	Male	1.78	79	10.5
16	21	Male	1.85	68	10.5
17	21	Female	1.63	68	8.5
18	23	Female	1.65	70	8.5
19	19	Male	1.85	61	10.5
20	21	Male	1.73	73	10.5
21	22	Female	1.73	68	8.5
22	22	Male	1.8	66	10.5
23	22	Female	1.63	75	8.5
24	21	Female	1.78	61	8.5
25	20	Female	1.68	59	8.5
26	21	Female	1.68	63	8.5
27	20	Female	1.68	66	8.5
28	21	Female	1.65	63	8.5
29	22	Male	1.91	86	10.5
30	20	Female	1.57	51	8.5
31	21	Female	1.68	63	8.5
32	21	Male	1.83	66	10.5
33	21	Female	1.68	68	8.5
34	20	Female	1.57	52	8.5
35	20	Female	1.65	52	8.5
36	20	Female	1.68	60	8.5
37	21	Female	1.63	68	8.5

**Table 3:** Number of FSR sensors and their effect on RMSE.

Test	Sensors On	Sensors Off	Total Sensors	L5S1 RMSE
1	S2, S5	S1, S3, S4, S6	4	3.21
2	S3, S4, S5, S6	S1, S2	8	2.30
3	S1, S3, S4, S6	S2, S5	8	2.15
4	S1 and S2 combined	None	10	1.83
5	S1, S2, S3, S5, S6	S4	10	0.95
6	S1, S2, S3, S4, S5, S6	None	12	0.30
